# Elements of chronic disease management service system: an empirical study from large hospitals in China

**DOI:** 10.1038/s41598-022-09784-8

**Published:** 2022-04-05

**Authors:** Shuzhen Zhao, Renjie Du, Yanhua He, Xiaoli He, Yaxin Jiang, Xinli Zhang

**Affiliations:** 1 West China School of Nursing/Outpatient Department, West China Hospital, Sichuan University, Chengdu, 610041 China; 2 Business School of Sichuan University, Chengdu, 610065 China

**Keywords:** Health care, Computer science

## Abstract

At present, more patients suffer from multiple chronic diseases. However, the hospital's existing chronic disease management is carried out according to the department. This means that a patient needs to go to more than one department for a chronic disease treatment. Therefore, this study proposes 6 dimensions (organizational management, medical service support, medical service, community alliance, self-management support, management information system) and 36 questions, to help evaluate the current chronic disease management system in China's large third-class hospitals. In this study, 143 survey samples from doctors and nurses were collected. A principal component analysis was used to extract three key elements of chronic disease management service delivery system (service management organization, management information system, medical core service). Then, multiple regression was used to establish the relationship model between the overall performance of the system and the main elements. Three key service nodes of the system (medical specialist support, patient tracking management and personalized intervention) were determined according to the weight of the regression model. The regression coefficients of the above three main elements show a similar impact on the overall performance of the system, but the key service nodes under each major element have relative differences, including medical specialist support, patient tracking management and personalized intervention. Finally, to establish a chronic disease management system with multiple departmental continuous care for chronic diseases, it is necessary to improve the chronic disease management system from three aspects of medical specialty support, patient tracking management and personalized intervention. This paper proposes corresponding improvement strategies.

## Introduction

Chronic disease is a general term for diseases with insidious onset, long incubation period, long and slow course of disease, delayed healing, lack of exact biological evidence, and unclear indication of “cure”^1^. Chronic diseases such as cardiovascular diseases, cancer, chronic respiratory disease, and diabetes are important restrictive factors for the improvement of human health and life expectancy. Chronic diseases account for 85% of all deaths in China^2^. It is predicted that by 2030, the total number of chronic disease-related deaths will rise to 70% of the total deaths worldwide (Action of Healthy China (2019–2030)^3^. Currently, an increasing number of patients have more than two chronic diseases at the same time^4–6^. Patients with multiple chronic diseases need to go to different departments in the process of chronic disease management in the hospital. Different departments need to review different case records, resulting in increased time cost and inconvenience to patients, which requires co-controlled. However, most of the existing management plans of chronic disease only focus on the control of one chronic disease, therefore, other chronic diseases are not being co-controlled.

Chronic disease management^7^ refers to chronic non-communicable diseases and their risk factors for regular and continuous testing, evaluation and comprehensive intervention in the management of medical behavior and process, main content, including early screening for slow disease, slow disease risk prediction, early warning and comprehensive intervention, and the integrated management of the patient group, and evaluation of the effect of slow disease management Therefore, chronic disease management can improve the quality of life of patients with chronic diseases.

Chronic disease management was first proposed by the American scholar Wagner (1998), based on the joint intervention of patients, and medical workers and medical policies, and which requires six elements: Community resources and policy support; health systems; data management of clinical information systems; design of a health service delivery system (team member tasks, follow-up plan development; joint decision making; and patient self-management). With the World Health Organization's expansion and extension of CCM in 2002 (World Health Organization^8^), the Innovative Care for the Chronic Conditions (ICCC) is proposed, and which consists of three levels: Micro level (patients and their families), intermediate level (health care institutions and communities); and macro level (policy and financial resource mobilization). In 2011, the United States launched a project known as Improve Chronic Illness Care^9^ (ICIC), which mainly supplemented five viewpoints: (1) Adding patient safety related content in the health system,(2) The design of the health service delivery system should consider the patient's educational level and ideology; (3) Adding case management to the design of the health service supply system; (4) Increasing care coordination in data management of health systems and clinical information systems; and (5) Providing community resources and a policy support system to supplement the community related policies. The integration of medical care with health service delivery systems enables patients with chronic diseases to receive timely diagnosis and treatment in any setting. Subsequently, some basic reference models for CDM were derived to help medical staff collaborate to make CDM plans^10^.

Since 2010, the above recommendations were integrated in the chronic disease management services of 15 departments of in West China Hospital of Sichuan University, including dermatology, rehabilitation, and oncology, and with a total of 46 service projects. In 2020, the number of chronic disease services in West China Hospital of Sichuan University reached 3855. At present, many patients with chronic disease may suffer from more than one chronic disease^11,12^, and the chronic disease management system of each department in West China is relatively independent. Therefore, in the chronic disease management of patients with multiple chronic diseases, the system information is complicated and messy, which severely affects the effective management of patients with chronic diseases. How to explore and develop an efficient model of chronic disease management that is suitable for West China Hospital of Sichuan University is the theme of this study.

**Figure 1:**
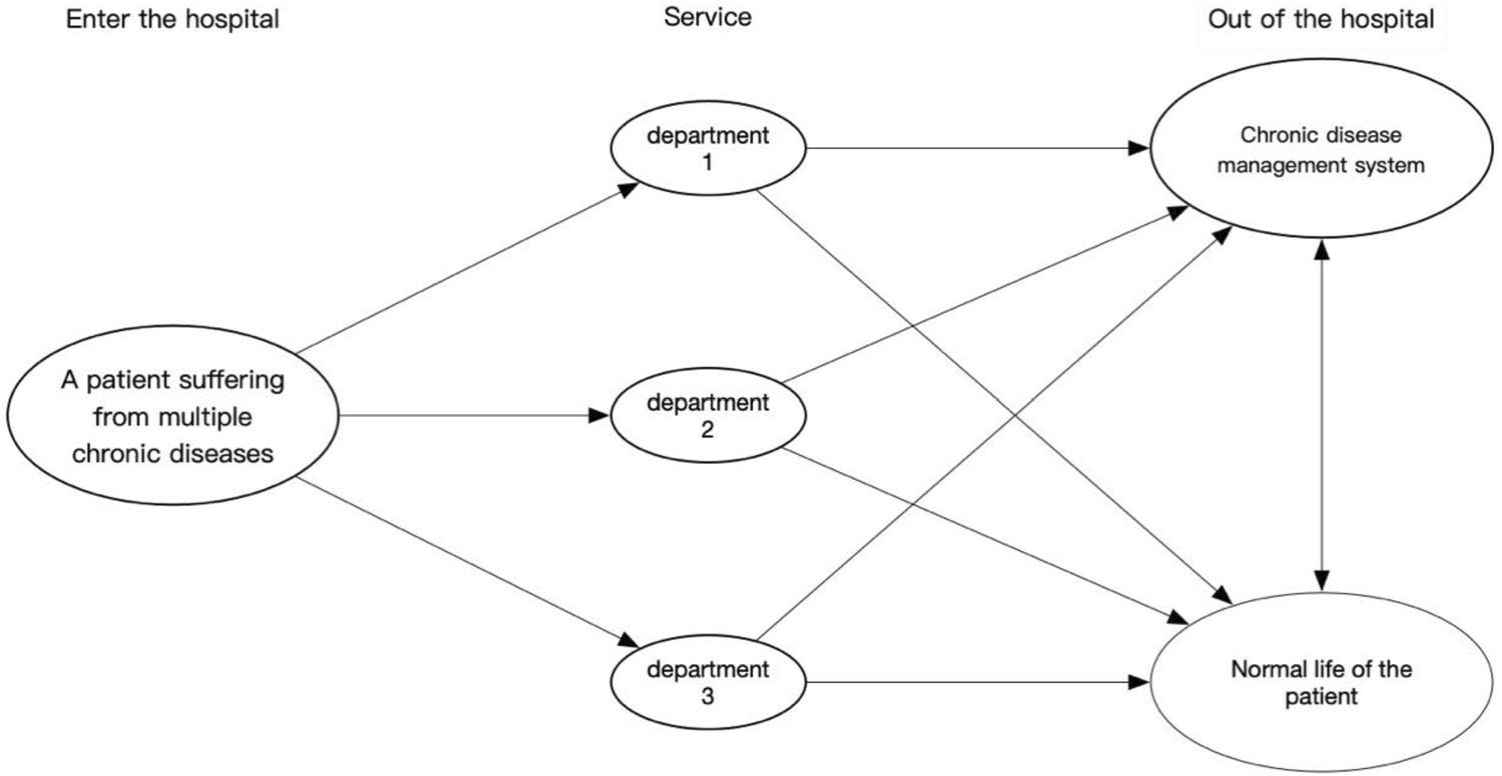
Schematic representation of chronic disease management in large hospitals. The arrows represent how patients with multiple diseases are transferred to different departments. Coordinated treatment by multiple departments: The arrows from the 3 department to the chronic disease management system indicate: after treatment, each department will input the patient's treatment into the chronic disease management system; The arrows from the 3 department to the patient's normal life indicate that the patient is discharged from hospital; The bidirectional arrow from chronic disease management system to patients' normal life indicates that patients input their daily physical conditions into the system, and the system feedback corresponding services according to patients' conditions.

This paper proposes the adoption of a unified chronic disease management mode for multiple chronic diseases and in multiple departments, which will provide a continuous care management mode of multiple departments and multiple chronic diseases (see Fig. 1). In this mode, different departments can coordinate management according to patients' various chronic diseases. Based on the existing chronic disease management service system of West China Hospital, this study evaluates the chronic disease management service system from six dimensions: Management organization; medical service support; medical service; community alliance; self-management support; and management information system.

From the service organizational perspective, the management of the chronic disease system requires the involvement and coordinated communications between nurses, specialists, and pharmacists in different departments. For example, specialists should regularly follow up patients and make accurate diagnosis according to the physical indicators based on the patient uploads to the management system^13^. Pharmacists provide guidelines for patients to take their medicines based on the specialist's diagnosis. In addition, when the continuous care of nurses is improved, the quality of chronic care can be improved^14^.

From the perspective of medical service support, improving the clinical competence of nurses can reduce nursing barriers and improve nursing quality^15^. The management of chronic disease relies on the support of a professional team. To cultivate an efficient professional team that manages chronic disease and allows patients to participate in the development and maintenance of a team-based chronic disease management, chronic disease management can be continuously improved, and therefore, the efficiency of the whole management can be improved^16^.

From the perspective of medical services, it refers to a continuous nursing that provides coherent, logical, and timely nursing for individuals^17^. The improvement of the quality of continuous nursing is helpful in improving the health level of patients with chronic diseases^18^. Patients seeking the support of professional nursing staff can reduce the probability of chronic disease deterioration that can be caused by an unhealthy lifestyle^19^.

From the perspective of community alliance, an appropriate health education can encourage patients to accept and use smart phones that support COPD patients' self-management. WeChat may play an important role in improving patient compliance and psychological distress^20^. During the COVID-19 epidemic, some patients with chronic diseases did not know how to properly use drugs, and community pharmacists provided services such as drug dispensing, consultation and referral, chronic disease management, safe infusion use, patient education, home nursing guidance, and psychological support in a variety of ways to ensure the safe use of drugs by the patients^21^. Community pharmacists play an important role in chronic disease management^22^. Latinos are the largest minority group in the United States, with higher rates of certain chronic diseases, and Latino community health workers have succeeded in improving the health status of their communities^23^.

From the perspective of patients' self-management behavior, self-management has become the main mode of chronic disease management and improving its strategy can also enhance the clinical outcomes of patients with chronic disease^24^. Chronic disease self-management support is making usage of patients' life goals and social roles to create patients’ self-management and obtain a better management effect.

From the perspective of cognition, the effect of chronic disease management depends on the cognition of patients^25^. A study of diabetic retinopathy^26^ found that people with diabetes had a low level of awareness of the risk factors associated with diabetic retinopathy, and thus, the chronic disease management system did not work well.

From the perspective of the management of the information system for chronic diseases, the continuous development of information and communication technology and artificial intelligence technology, allows the combination of the electronic health management information system for diagnosis with the treatment suggestion system to realize remote monitoring of patients, which improve the service efficiency and quality^27^. Online consultation can not only reduce the cost of medical appointment and health monitoring, but also reduce the patients’ difficulty in obtaining treatment and their overall treatment burden^28^. To control the development of chronic diseases, China is constantly improving the community health service information system^29^. For instance, Beijing introduced the Australian chronic disease management system for diabetes and evaluated the treatment effect through screening data from the ICDMS database. The research showed that the patients' physical indicators gradually became normal after the implementation of the chronic disease management system.

Finally, the hospital establishes the process of chronic disease management. The standardized service process of chronic disease management is as follows: (1) Establishment of health records from the time the patients find out about their condition and their follow up through the management system that diagnoses and classifies the disease, to the professional doctors who evaluate the results; (2) Carrying out hospital treatment, medicine, and psychological guidance; and (3) Daily monitoring of the patient's physical condition after discharge.

The management of chronic disease management requires a multi-dimensional coordination of organization management, medical service and support, patient self-management, community alliance, and management information system. There are significant differences in various chronic diseases, but from the management perspective, the same service system for chronic disease management can be shared. It can standardize the diagnosis and treatment between various departments and individually adjust the chronic disease management program to realize the integrated management of multiple chronic diseases. The advantage of co-controlled is that doctors from different departments can simultaneously consult patients' conditions online or off-line, or different doctors can learn about other doctors' descriptions of patients' conditions in the system, which is convenient for doctors to make efficient diagnosis.

This study focuses on investigating the continuous care management model of multiple departments and chronic diseases in West China Hospital of Sichuan University by performing an empirical study of chronic disease management service system based on several perspectives associated with chronic disease management. This approach will help extract the components of the chronic disease management service system, determine the key service nodes of each element, and put forward suggestions for the development of chronic disease management service system that can further optimize the chronic disease management service system of West China Hospital of Sichuan University, and provide better services for patients with chronic diseases.

The organization of the article includes an introduction, a method section, including questionnaire design, descriptive analysis, principal component analysis, and regression analysis, a results’ section which identifies the main system elements and key service nodes, suggestions’ section focused on providing recommendations for the improvement of chronic disease management service delivery system, and a summary.

## Method

### Questionnaire design

Based on the management practice of chronic disease and literature of large Hospitals in China (Wagner, E.H.), this study designed a questionnaire with 36 questions from six dimensions, including organizational management, medical service support, medical service, patient self-management, community communication, and management information system. The last question was the overall satisfaction evaluation of the system (Table 1).

**Table 1:** Dimension and variable design questionnaire.

Dimensionality	Number	Variable	Questionnaire items	Variable
Organizational management	${M}_{1}$	${X}_{1}$	1.1 Department of chronic disease management job involvement	${x}_{11}$
			1.2 Department of setting up chronic disease management goals	${x}_{12}$
			1.3 Department of chronic disease management incentives and performance	${x}_{13}$
			1.4 Head of department management attitude to chronic disease management	${x}_{14}$
			1.5 Organizational structure of departmental chronic disease management	${x}_{15}$
Medical service support	${M}_{2}$	${X}_{2}$	2.1 Support for chronic disease management training of medical staff in chronic disease management	${x}_{21}$
			2.2 Query of knowledge about chronic disease management	${x}_{22}$
			2.3 Chronic disease management service information system assistance in decision-making	${x}_{23}$
Medical services	${M}_{3}$	${X}_{3}$	3.1 Nursing care	${x}_{31}$
			3.2 Medication guidance	${x}_{32}$
			3.3 Counseling	${x}_{33}$
			3.4 Appointment for follow-up	${x}_{34}$
			3.5 Comorbidity management	${x}_{35}$
			3.6 Green channel for registration	${x}_{36}$
			3.7 Medication supervision	${x}_{37}$
			3.8 Chronic disease management service process standard	${x}_{38}$
			3.9 Departments' understanding of patients' needs	${x}_{39}$
			3.10 Response of departments to patients' needs	${x}_{3. 10}$
			3.11 Condition monitoring	${x}_{3. 11}$
			3.12 Internet prescription	${x}_{3. 12}$
Community alliance	${M}_{4}$	${X}_{4}$	4.1 Cooperation between departments and communities in chronic disease management	${x}_{41}$
			4.2 Departmental mobilization of family members to participate in patient management	${x}_{42}$
			4.3 Interdepartmental cooperation within the hospital in chronic disease management	${x}_{43}$
			4.4 The department cultivates patients who are active in the group	${x}_{44}$
			4.5 Cooperate with research team in chronic disease management	${x}_{45}$
Self-management support	${M}_{5}$	${X}_{5}$	5.1 Department of patient work education	${x}_{51}$
Management information system	${M}_{6}$	${X}_{6}$	6.1 Information support system for organization design of chronic disease management	${x}_{61}$
			6.2 Information support system for decision support of chronic disease management	${x}_{62}$
			6.3 Information system tips for chronic disease management	${x}_{63}$
			6.4 Appointment and green channel system support for the registration of patients with chronic diseases	${x}_{64}$
			6.5 Information system monitoring and early warning of chronic disease for patients	${x}_{65}$
			6.6 Chronic disease information uploaded from the community	${x}_{66}$
			6.7 Chronic disease patients' condition information is transmitted to the community	${x}_{67}$
			6.8 Systematic description of the treatment process of patients with chronic diseases in hospital	${x}_{68}$
			6.9 The information system assists medical staff to complete the education, consultation, and guidance of patients with chronic diseases	${x}_{69}$
			6.10 The information system receives patient self-management feedback	${x}_{6. 10}$
Overall evaluation	${M}_{7}$	$Y$	7.1 Overall evaluation of chronic disease management	$y$

### Data statistics and methods

Excel 2016 was used for data sorting, and the mean value of answers to each question was respectively collected. The SPSS software was used for statistical analysis of data reliability analysis. SPSS was used for factor validity test and the principal components were removed. At last, regression analysis was performed with global evaluation and principal component. Regression analysis satisfies normal distribution, independence of variables and logical relationship between independent variables and dependent variables.

### Ethical approval and informed consent

The doctors and nurses who surveyed for the study knew and agreed that the data from the questionnaire would be used to write this paper. All the participants in this paper were informed. The study was approved by The Ethics Committee of West China Hospital and informed consent was obtained. In this study, all adopted methods were permitted by Sichuan University. All the used methods were conducted according to relevant guidelines and regulations, and scientific rigor was always maintained.

## Results

### Preliminary sample analysis

Firstly, doctors and nurses who are service providers of chronic disease management in West China Hospital of Sichuan University were enrolled and requested to respond to the questionnaire. A total of 143 questionnaires were collected. The reliability and validity of the data obtained from the questionnaire are analyzed in Tables 2 and 3, respectively. Through Cronbach's Alpha and KMO, we know that the data have good reliability and validity.

**Table 2:** Reliability statistics scale.

Cronbach's alpha	Cronbach's alpha based on standardized items	Number of items
0.971	0.971	143

**Table 3:** Validity analysis.

Kaiser–Meyer–Olkin measure of sampling adequacy	0.881
Approx. Chi-square	5641.337
df	630
Sig	0.000

According to the survey’s data, the overall evaluation score of chronic disease management service system had a maximum of 5 points and a minimum of 2 points and with an overall mean of 4.026 and a mean variance of 0.696. The organization’s management, medical service, patients' self-management, and community alliance dimensions, were higher than 4.026, while the mean value of medical service support and management information system was less than 4.026. The highest score of service node was ${x}_{14}$ and ${x}_{11}$ in organizational design, and the lowest score was ${x}_{6. 10}$ in management information system (Table 4).

**Table 4:** Variable mean and variance.

Dimension	Variable	Mean	Variance	Dimension	Variable	Mean	Variance
${M}_{1}$	${X}_{1}$	4.276	0.584		${x}_{3. 11}$	4.063	0.524
	${x}_{11}$	4.465	0.545		${x}_{3. 12}$	4.113	0.565
	${x}_{12}$	4.303	0.408	${M}_{4}$	${X}_{4}$	4.092	0.638
	${x}_{13}$	4.225	0.752		${x}_{41}$	4.014	0.535
	${x}_{14}$	4.528	0.376		${x}_{42}$	4.120	0.711
	${x}_{15}$	3.859	0.839		${x}_{43}$	4.049	0.554
${M}_{2}$	${X}_{2}$	4.011	0.745		${x}_{44}$	4.092	0.745
	${x}_{21}$	3.852	0.942		${x}_{45}$	4.183	0.643
	${x}_{22}$	3.767	0.657	${M}_{5}$	${X}_{5}$	4.042	0.519
	${x}_{23}$	4.415	0.637		${x}_{51}$	4.042	0.519
${M}_{3}$	${X}_{3}$	4.183	0.663	${M}_{6}$	${X}_{6}$	3.701	0.824
	${x}_{31}$	4.444	0.655		${x}_{61}$	3.768	0.685
	${x}_{32}$	4.106	0.728		${x}_{62}$	3.690	0.510
	${x}_{33}$	4.303	0.690		${x}_{63}$	3.838	0.727
	${x}_{34}$	4.169	0.788		${x}_{64}$	3.887	0.649
	${x}_{35}$	4.148	0.732		${x}_{65}$	3.613	0.773
	${x}_{36}$	4.070	0.784		${x}_{66}$	3.627	1.206
	${x}_{37}$	4.289	0.529		${x}_{67}$	3.613	1.054
	${x}_{38}$	4.056	0.532		${x}_{68}$	3.704	0.997
	${x}_{39}$	4.366	0.697		${x}_{69}$	3.697	0.830
	${x}_{3.10}$	3.887	0.734		${x}_{6.10}$	3.570	0.808

### Analysis of the principal component

Due to chronic disease management service system that involved multiple dimensions, there was an inevitable collinearity among variables within the dimensions. A principal component analysis was performed, and the 36 variables in the sub-problem were dimensionality reduced to extract the principal components. The principal component analysis was performed using SPSS 26.0.

**Figure 2:**
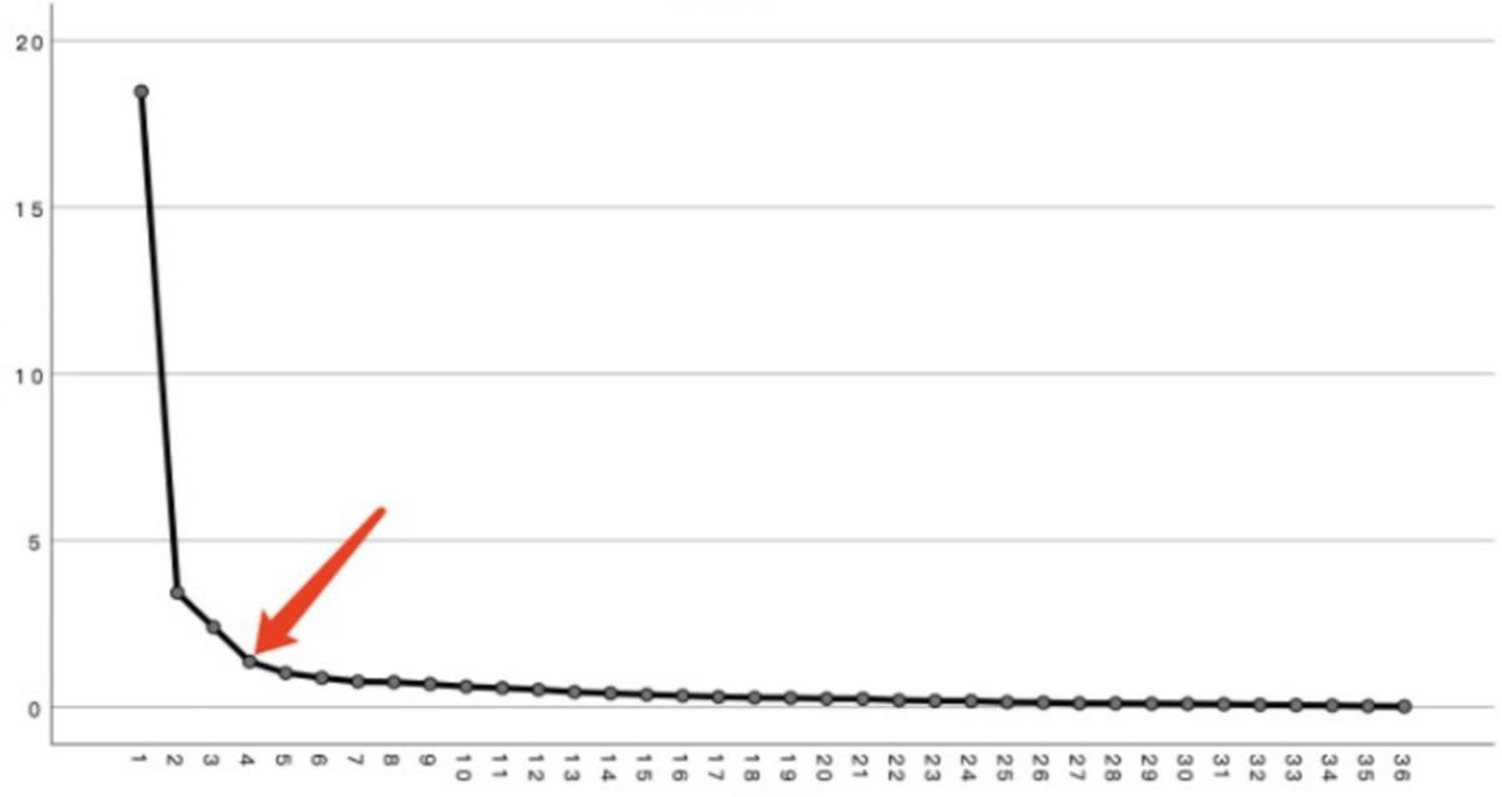
Scree plot. *Note* The vertical axis is the eigenvalue and the horizontal axis is component.

In Table 5, the eigenvalues of the five factors were greater than 1, and their total interpretation rate was 73.140%, and their corresponding interpretation rates were 51.303%, 9.538%, 6.667%, 3.796%, and 2.836%. The results of the Scree Plot (Fig. 2) showed when starting from the fourth factor, the difference in characteristic values of the subsequent factors was small, and therefore, 3 principal components were finally extracted (see Table 6) on the judgment condition that the interpretation rate of the principal components is greater than 5%.

**Table 5:** Eigenvalues and variance percentages of the five factors.

Component	Total	Of variance (%)	Cumulative (%)
1	18.469	51.303	51.303
2	3.434	9.538	60.841
3	2.400	6.667	67.508
4	1.366	3.796	71.304
5	1.021	2.836	73.140

**Table 6:** Three principal component eigenvalues and variance percentages.

Component	Total	Of variance (%)	Cumulative (%)
1	18.469	51.303	51.303
2	3.434	9.538	60.841
3	2.400	6.667	67.508

The data was rotated, and variables with coefficients of < 0.55 were deleted from the table to obtain factor rotation matrices under three principal components, which were arranged according to variable coefficients under each principal component (Table 7).

**Table 7:** Rotated component matrix.

Variable	Component 1	Component 2	Component 3	Variable	Component 1	Component 2	Component 3
${x}_{22}$	0.812			${x}_{42}$			
${x}_{23}$	0.727			${x}_{68}$		0.851	
${x}_{45}$	0.717			${x}_{67}$		0.845	
${x}_{14}$	0.717			${x}_{6.10}$		0.831	
${x}_{43}$	0.714			${x}_{66}$		0.827	
${x}_{13}$	0.690			${x}_{65}$		0.826	
${x}_{3.10}$	0.665			${x}_{69}$		0.775	
${x}_{3. 11}$	0.663			${x}_{63}$		0.735	
${x}_{38}$	0.653			${x}_{62}$		0.717	
${x}_{12}$	0.635			${x}_{61}$		0.635	
${x}_{39}$	0.633			${x}_{64}$		0.582	
${x}_{15}$	0.595			${x}_{34}$			0.864
${x}_{51}$	0.591			${x}_{32}$			0.805
${x}_{21}$	0.590			${x}_{35}$			0.802
${x}_{41}$	0.564			${x}_{31}$			0.800
${x}_{11}$	0.563			${x}_{37}$			0.786
${x}_{3.12}$	0.556			${x}_{36}$			0.764
${x}_{44}$	0.552			${x}_{33}$			0.755

### Regression analysis

The three principal components were respectively expressed as *F*_1_, *F*_2_, and *F*_3_, and the dependent variable Y was used as the overall satisfaction evaluation of the chronic disease management service delivery system. A multiple regression analysis was also conducted with SPSS 26.0 and the regression coefficient structure of the three system elements is shown in Table 8.

**Table 8:** Regression coefficient.

Components	B	SE	t	$p$
Constant	4.035	0.37	108.277	***
${F}_{1}$	0.375	0.37	10.033	***
${F}_{2}$	0.334	0.37	8.937	***
${F}_{3}$	0.337	0.37	9.009	***

**Means a significant test that passes 0.05, and ***means a significant test that passes 0.01.

As a result, the regression equation was:

$$Y=4.035+0.375{F}_{1}+0.334{F}_{2}+0.337{F}_{3}$$

### Summary

The service delivery system can be summarized into three component elements that are respectively named as service organization management, management information system, and medical core service (Table 7). There was no significant difference in the regression coefficients of each component, indicating that each system element has the same importance in the satisfaction performance of the chronic disease management service delivery system (Table 8).

In terms of service nodes, the ranking was based on the degree of influence of node variables on system elements. In each dimension, the naming and grading of service system by node variables, under the three system elements, are shown in Table 9.

**Table 9:** Chronic disease management service system elements and average scores of service nodes under the elements.

Rotated component coefficients	Organization management		Management information system		Medical core service	
	Variable	Definition	Variable	Definition	Variable	Definition
0.8–0.9	${x}_{22}$	Specialized services (3.767)	${x}_{68}{x}_{67}{x}_{6. 10}{x}_{66}{x}_{65}$	Patient management (3.625)	${x}_{34}{x}_{32}{x}_{35}{x}_{31}$	Personalized intervention (4.217)
0.7–0.8	${x}_{23}{x}_{45}{x}_{14}{x}_{43}$	Hospital union (4.293)	${x}_{69}{x}_{63}{x}_{62}$	Information technology support (3.742)	${x}_{37}{x}_{36}{x}_{33}$	Special care (4.221)
0.6–0.7	${x}_{13}{x}_{3. 10}{x}_{3. 11}{x}_{38}{x}_{12}{x}_{39}$	Service specification (4.15)	${x}_{61}$	Information system application (3.77)		
0.5–0.6	${x}_{15}{x}_{51}{x}_{21}{x}_{41}{x}_{11}{x}_{3. 12}{x}_{44}$	Coordination of organization (4.062)	${x}_{64}$	Green channel management (3.88)		

The values in brackets indicate the mean of the system dimensions.

Among them, specialized service, patient management, and personalized intervention, are the key nodes, and the scores were 3.767, 3.6254, and 4.217, respectively. When using 4.026 as the baseline, personalized intervention had the highest value in the existing chronic disease management service system.

## Discussion

### Concept diagram of patient management system

**Figure 3:**
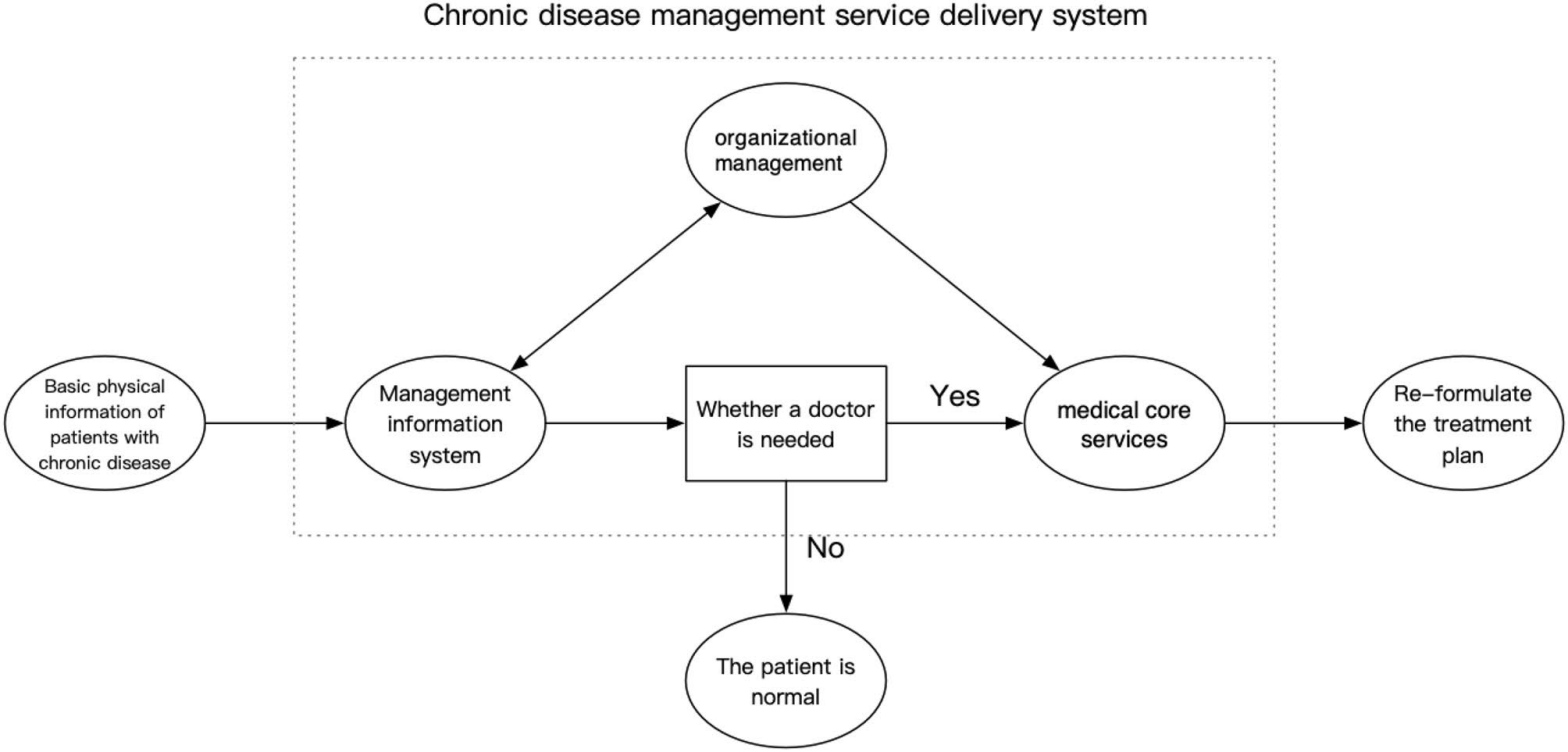
Concept diagram of chronic disease patient management system. For the whole chronic disease management delivery system, the patients input their physical status (such as blood pressure, blood sugar, etc.) into the management information system every day. According to the patient's physical data, the management information system determines whether a doctor is needed to intervene in the diagnosis and treatment. If not, it means that the patient's condition is in a normal level and can be self-managed. If a physician intervention is needed, the patient's treatment plan will need to be re-customized, such as whether to be hospitalized, changing the dosage of drugs, and improving the diet.

According to the results of the empirical research, the chronic disease relationship service delivery system can be summarized into three system elements, including organizational management, management information system, and core medical services. Organizational management can integrate service resources and standardize the service processes, providing a basis for the design of management information system. Meanwhile, the management information system can evaluate the rationality of service organization and management, both of which can provide support for core medical services. The concept diagram of chronic disease management service delivery system is shown in Fig. 3.

### Suggestions for the improvement of chronic disease patient management system

According to the research results, the existing distributed chronic disease management service delivery system for continuous care of multi-chronic diseases and multi-departments in West China Hospital of Sichuan University, needs to be improved from the key node of specialized service and the dimension of management information system, using the following suggestions:

1. Establishment and improvement of the standard for chronic disease management and service
  Through the introduction of chronic disease management evaluation policies by relevant national departments, various expert chronic disease management guidelines and detailed rules are formulated to provide clinical medical staff with professional chronic disease related knowledge and preventive intervention measures to ensure the quality of chronic disease management.
2. Establishment of a unified information platform for patients with chronic diseases
  The current information level is still outdated and there is a need to construct an efficient chronic disease management model that can resolve problems such as inadequate long-term follow-up monitoring and intervention for patients. Chronic disease managers need to fully evaluate the health conditions and needs of patients to develop a comprehensive personalized management plan and efficiency in managing chronic disease patients. Building a unified information system to share information on the patients' diagnosis and treatment in each department and help medical staff in their understanding of the patients’ disease progression, to improve the efficiency of chronic disease management.
3. Strengthening the process management of patients' health data
  Patients with chronic disease have many complications and a long course of disease, which will generate a large amount of health data. However, the current follow-up mechanism is not perfect, the follow-up system is weak, and a large part of patients are followed. The health data of patients cannot be fully grasped by medical staff, which can easily lead to the disconnection of chronic disease management process. Multi-channel collection and feedback of patients' health data should be performed, a set of standard process for multi-channel management of patients with chronic diseases' health data should be developed, and the working process should be continuously optimized to improve work efficiency.

## Conclusion

This study based on the management of 143 slow disease by various departments through the investigation and analysis of the service delivery system that was carried out based on the statistics of a questionnaire, sample sampling, the descriptive and explanatory analysis of the slow disease management service system that is based on empirical research, the construction of additional elements of the slow delivery system for the management of patients and with a figure, formation of the slow disease service management of medical practice advice. Therefore, this model has a practical value in improving the quality of chronic disease management service delivery system in large hospitals.

Compared with the literature^30,31^, this model added more service system dimensions, including six aspects of organizational management, medical service support, medical service, patient self-management, community alliance, and management information system. Meanwhile, three system elements affecting the service efficiency and key service nodes under the elements, were found through principal components. The studied service objects of the chronic disease management service delivery system studied provides solutions for patients suffering from both a single and multiple chronic diseases.

Cost is not considered in this paper for the time being, and corresponding cost discussion will be added in future research.

The research results provide ideas for the coordination of multiple departments. Although the empirical research object of this study is typical, the number of samples collected is limited, and the research results still need to be further verified in practice.
